# Nuclear-accumulated SQSTM1/p62-based ALIS act as microdomains sensing cellular stresses and triggering oxidative stress-induced parthanatos

**DOI:** 10.1038/s41419-018-1245-y

**Published:** 2018-12-13

**Authors:** Takuya Noguchi, Midori Suzuki, Natsumi Mutoh, Yusuke Hirata, Mei Tsuchida, Sayoko Miyagawa, Gi-Wook Hwang, Junken Aoki, Atsushi Matsuzawa

**Affiliations:** 1 Laboratory of Health Chemistry, Graduate School of Pharmaceutical Sciences, Tohoku University, Aoba-6-3, Aramaki, Aoba-ku, Sendai, 980-8578 Japan; 2 Laboratory of Molecular and Biochemical Toxicology, Graduate School of Pharmaceutical Sciences, Tohoku University, Aoba-6-3, Aramaki, Aoba-ku, Sendai, 980-8578 Japan; 3 Laboratory of Molecular and Cellular Biochemistry, Graduate School of Pharmaceutical Sciences, Tohoku University, Aoba-6-3, Aramaki, Aoba-ku, Sendai, 980-8578 Japan

## Abstract

Aggresome-like induced structures (ALIS) have been described as ubiquitinated protein-containing aggresomes transiently formed in response to various stresses. In this study, we provide evidence that ALIS composed of SQSTM1/p62 act as a key determinant of oxidative stress-induced parthanatos, which is newly discovered and distinct from regular programmed cell death. Interestingly, we first found that chemical stresses induced by particular chemical drugs, such as several cephalosporin antibiotics, cause oxidative stress-mediated parthanatos, accompanied by the ALIS formation. Blocking the ALIS formation potently suppressed the parthanatos, and p62 knockout cells exhibited the attenuated ALIS formation and high resistance to parthanatos. Moreover, we also found that the redox-sensing activity of p62 is required for nuclear accumulation of the p62-based ALIS, resulting in the induction of parthanatos. Together, our results demonstrate unexpected functions of p62 and ALIS as cell death mediators sensing oxidative stress, and thus uncover a novel mechanism whereby p62 mediates parthanatos.

## Introduction

ALIS refer to ubiquitin-containing aggresomes that function as protein storage compartments for the sequestration of misfolded proteins, which are generated by various cellular stresses^1^. It has been reported that a wide variety of stresses, including amino acid starvation, virus infection, endoplasmic reticulum stress, lipopolysaccharide (LPS), and oxidative stress, induce the ALIS formation^1–5^. Inhibition of either proteasome or autophagy enhances ALIS formation and interferes with ALIS clearance, indicating that, compared with other intracellular aggresomes, ALIS are transient and reversible protein aggregations that are associated with autophagic or proteasomal activity^1^. ALIS formation reflects cellular stresses, and therefore, ALIS are supposed to be microdomains sensing cellular stresses. Although the molecular mechanisms of the ALIS formation are poorly characterized, the ubiquitin-binding protein p62 (also known as sequestosome-1, SQSTM-1 or A170) has been emerged as a key component of ALIS^2,6,7^. p62 was initially identified as a binding partner of atypical protein kinase C (PKC)^8^, and subsequent studies have revealed the multifunctional roles of p62 as a signaling adaptor and autophagic cargo receptor^9–11^. At a cellular level, p62-containing aggregates are observed under various stress conditions, and p62 is required for the ALIS formation that mediates degradation of ubiquitinated proteins by autophagy^2,6,12^. On the other hand, in neurodegenerative diseases, p62 is found in inclusion bodies containing polyubiquitinated protein aggregates, such as Lewy bodies in Parkinson disease, Huntingtin aggregates in Huntington disease, and neurofibrillary tangles in Alzheimer disease^13–16^. In liver diseases, such as alcoholic and nonalcoholic steatohepatitis, Mallory body in hepatocytes also includes large amounts of p62^16^. Together, these findings indicate a pathologically close link between p62 and the diseases associated with protein aggregates, including ALIS.

Mammalian cells are continuously exposed to reactive oxygen species (ROS), which are generally counteracted by the endogenous antioxidant machinery, including Kelch-like ECH-associated protein-1 (Keap1)-NF-E2-related factor-2 (Nrf2) system^17–19^. Nrf2 is a transcription factor critical for the maintenance of cellular redox homeostasis^19^. Under resting conditions, Nrf2 is ubiquitinated by the Keap1-Cullin3 ubiquitin ligase complex and is routinely degraded by the 26s proteasome, whereas, upon oxidative stress conditions, the activity of the ubiquitin ligase is blocked through the modification of cysteine residues in Keap1, resulting in Nrf2 stabilization and activation^20^. Nrf2 then translocates to the nucleus where it exerts its transcriptional activity through binding to the antioxidant response element (ARE) that is a master regulator of antioxidant gene expression^21^. Interestingly, p62 harbors a Keap1 interacting region (KIR), which allows to participate in the regulation of the Keap1-Nrf2 system, and in fact, p62 can mediate the stabilization and activation of Nrf2^9^. More recently, it has been shown that p62 possesses oxidation-sensitive cysteines, and thereby can directly sense ambient redox status^22^. Therefore, p62 has emerged as a potential regulator of redox signaling.

On the other hand, once ROS generation exceeds the capacity of the antioxidant machinery, cells suffer so-called “oxidative stress”. Under oxidative stress conditions, death-inducing signals are frequently activated to eliminate damaged cells that may cause tumorigenic transformation^23^. Dysfunction of the signaling molecules that mediate oxidative stress-induced cell death is known to be a potential cause of many diseases, such as cardiovascular diseases, hepatitis, diabetes mellitus, neurodegenerative diseases, and cancer^24,25^. Thus, the induction of programmed cell death is an essential cellular response to oxidative stress. It has been demonstrated that apoptosis signal-regulating kinase 1 (ASK1)-thioredoxin (Trx) system functions as a sensor of oxidative stress that induces apoptotic cell death^26,27^. Meanwhile, accumulating evidence indicates that oxidative stress has an ability to induce diverse types of non-apoptotic cell death, including necroptosis, ferroptosis, pyroptosis, and parthanatos, in a context-dependent manner^28^. However, the molecular mechanisms governing cellular responsiveness to oxidative stress-induced cell death, which may be a key to understanding the pathogenesis of diseases associated with oxidative stress, remain poorly understood.

Parthanatos is one of the manner of non-apoptotic programmed cell death that is mediated by poly (ADP-ribose) polymerase-1 (PARP-1)^29–31^. Under physiological conditions, PARP-1 serves to control DNA repair and genomic stability^32^. On the contrary, under pathological conditions that cause hyperactivation of PARP-1 frequently mediated by severe genomic stress, PARP-1 stimulates the nuclear translocation of the mitochondrial-associated apoptosis-inducing factor (AIF), which causes large-scale DNA fragmentation and chromatin condensation, leading to cell death^33,34^. There is ample evidence to suggest that parthanatos is implicated in the pathogenesis of a wide variety of human diseases^30^. In particular, parthanatos is suggested to be involved in the pathological processes of the neurodegenerative diseases, such as Parkinson disease^35^.

In this study, we unexpectedly demonstrate that p62 stimulates parthanatos under oxidative stress conditions. The induction of oxidative stress-induced parthanatos requires the formation and nuclear accumulation of p62-based ALIS. Although ALIS are conceived as stress-induced protein storage compartments for substrates of the proteasome and autophagy, our results indicate that the p62-based ALIS serve as microdomains sensing oxidative stress and have important roles in signaling epicenters and mediators of oxidative stress-induced parthanatos. We actually observed as a pathophysiological phenomenon that chemical stresses generated by particular chemical drugs, such as several cephalosporin antibiotics, which have several side effects, triggered oxidative stress-mediated parthanatos through formation and nuclear accumulation of the p62-based ALIS. Thus, our results provide a novel function of p62 and an unexpected biological action of ALIS, which might be crucial for understanding the pathogenesis of p62- and ALIS-related human diseases, such as neurodegenerative diseases and alcohol- or chemical drugs-induced tissue damage, based on an unusual programmed cell death, parthanatos.

## Results

### The ALIS formation mediated by the cephalosporins commits to cell death

**Fig. 1:**
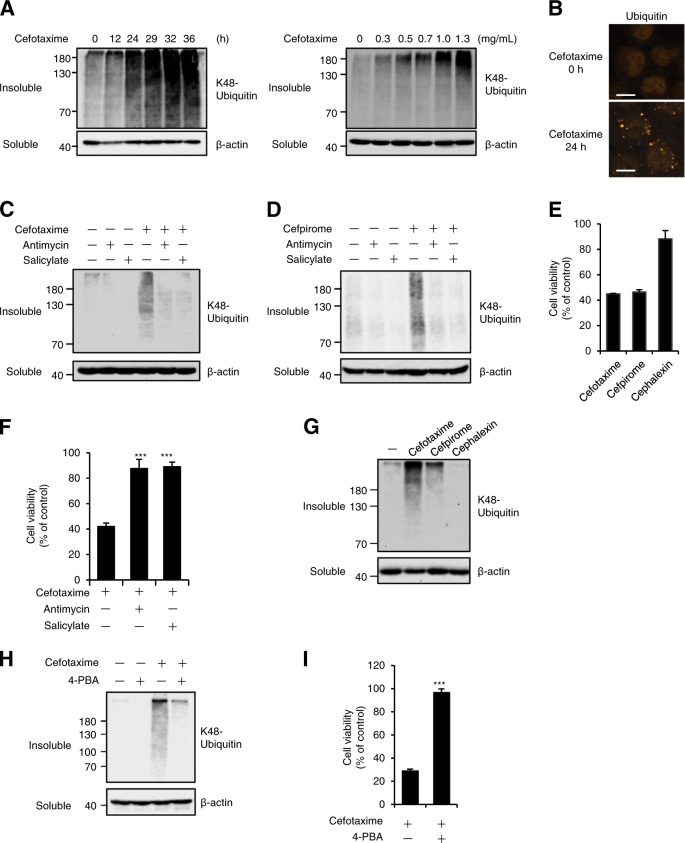
The ALIS formation mediated by the cephalosporins commits to cell death. **a** HT1080 cells were treated with 1 mg/ml cefotaxime for the indicated periods or with the indicated concentrations of cefotaxime for 36 h. The detergent-soluble and -insoluble fractions were subjected to immunoblotting with the indicated antibodies. **b** HT1080 cells were treated with 1 mg/ml cefotaxime for 36 h, and then performed immunofluorescence staining with ubiquitin antibody. Scale bar, 10 μm. **c**, **d** HT1080 cells were treated with the indicated reagents for 36 h, and then immunoblot analysis was performed with the indicated antibodies. **e** HT1080 cells were treated with the indicated reagents for 48 h, and then subjected to cell viability assay. Data shown are the mean ± SD. **f** HT1080 cells were treated with 1 mg/ml cefotaxime for 48 h with the indicated reagents, and then subjected to cell viability assay. Data shown are the mean ± SD. Significant differences were determined by one-way ANOVA, followed by Tukey–Kramer test; ***p < 0.001; **p < 0.01. **g** HT1080 cells were treated with the indicated reagents for 36 h, and then immunoblot analysis was performed with the indicated antibodies. **h**, **i** HT1080 cells were treated with the indicated reagents for 24 h (**h**) or 48 h (**i**), and then immunoblot analysis (**h**) or cell viability assay (**i**) was performed. Cefotaxime, Cefpirome, and Cephalexin (1 mg/ml), Antimycin (10 ng/ml), Salicylate (10 mM), 4-PBA (2 mM)

Generally, several chemical drugs and antimicrobial agents, such as cephalosporin antibiotics, give various cellular stresses to mammalian cells, which lead to sever medicinal side effects. However, the mechanisms underlying induction of various cellular stresses by chemical drugs and antimicrobial agents remain unclear. Interestingly, we found that several cephalosporin antibiotics exhibit cytotoxicity in mammalian cells, even though they are conceived as safe drugs and are commonly prescribed. To understand the mechanisms, we firstly investigated the cellular responses triggered by cephalosporin antibiotics. In this investigation, we first noticed that cefotaxime, a third-generation cephalosporin antibiotic, causes the aberrant aggregation of K48-linked polyubiquitinated proteins in the detergent-insoluble fraction of human fibrosarcoma HT1080 cell lysates in a time- and concentration-dependent manner, and observed an increase in immunofluorescent puncta of ubiquitinated proteins in cefotaxime-treated HT1080 cells (Fig. 1a, b). We also noticed that the detergent-insoluble aggregation was largely eliminated by co-treatment with antimycin or salicylate, known as macroautophagy inducers (Fig. 1c), and a similar result was clearly observed when HT1080 cells were treated with cefpirome, a fourth-generation cephalosporin antibiotic (Fig. 1d)^36,37^. These results suggest that the aggregation induced by the cephalosporins is the transient structure, which is discarded by the autophagic processes and whose features are similar to ALIS. We therefore investigated physiological roles of the cephalosporin-induced ALIS in cells.

When HT1080 cells were treated with the cephalosporins, both cefotaxime and cefpirome exhibited increased cytotoxicity that results in reduced cell viability, whereas cephalexin, a first-generation cephalosporin antibiotic, did not (Fig. 1e). Cefotaxime and cefpirome are different from cephalexin in structures of side chains from the basic skeleton β-lactam ring. These observations indicate that structures of side chains extended from the basic skeleton probably determine the cytotoxicity induced by the cephalosporins. Interestingly, co-treatment with antimycin or salicylate conferred a dramatic increase in cell viability (Fig. 1f), and cephalexin that showed little cytotoxicity in Fig. 1e failed to induce the ALIS formation (Fig. 1g). Moreover, 4-phenylbutyrate (4-PBA), which acts as a chemical chaperone and then suppresses protein aggregation, clearly suppressed cefotaxime-induced ALIS formation and cytotoxicity (Fig. 1h, i), in accordance with the previous report^38^. Collectively, these observations indicate an intimate link between the ALIS formation and increased cytotoxicity, and we speculated that the ALIS formation is responsible for cell death triggered by particular cellular stresses, including the cephalosporin-induced chemical stress.

### Oxidative stress is responsible for the ALIS formation and cell death induced by the cephalosporins

**Fig. 2:**
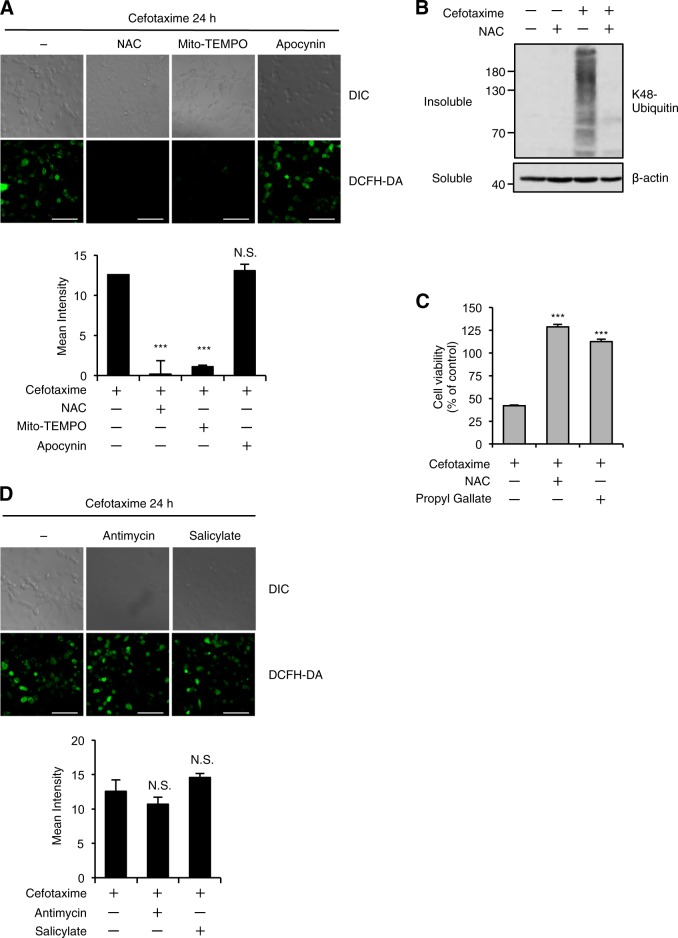
Oxidative stress is responsible for the ALIS formation and cell death induced by the cephalosporins. **a**, **d** HT1080 cells were treated with the indicated reagents for 24 h, and then treated with 10 μM DCFH-DA. Fluorescence images (upper panel) and intensity (lower panel) of HT1080 cells were acquired as described in the materials and methods section. Cell morphology was determined by Nomarski differential interference contrast (DIC) microscopy. Data shown are the mean ± SD. Significant differences were determined by one-way ANOVA, followed by Tukey–Kramer test; ***p < 0.001; N.S., not significant. Scale bar, 50 μm. **b** HT1080 cells were treated with the indicated reagents for 36 h, and then the detergent-soluble and -insoluble fractions were subjected to immunoblotting with the indicated antibodies. **c** HT1080 cells were treated with the indicated reagents for 48 h, and subjected to cell viability assay. Data shown are the mean ± SD. Significant differences were determined by one-way ANOVA, followed by Tukey–Kramer test; ***p < 0.001. Cefotaxime (1 mg/ml), NAC (1 mM), Mito-TEMPO (10 µM), Apocynin (100 µM), Propyl Gallate (20 µM), Antimycin (10 ng/ml), Salicylate (10 mM)

Accumulating evidence suggests that the bactericidal antibiotics cause mitochondrial dysfunction and oxidative stress in mammalian cells^39,40^. We therefore hypothesized that the cephalosporins cause oxidative stress, resulting in the induction of cell death. Microscopic analysis using the ROS indicator 2′, 7′-dichlorodihydrofluorescein diacetate (DCFH-DA) revealed that cefotaxime induces ROS generation, which is largely suppressed by co-treatment with antioxidants, such as *N*-acetylcysteine (NAC) and the mitochondria-targeted antioxidant Mito-TEMPO, but not apocynin that interferes with the function of the membrane-bound ROS generator NADPH oxidase, suggesting that the cephalosporins increase mitochondrial ROS generation (Fig. 2a). We also found that co-treatment with NAC clearly inhibited cefotaxime-induced ALIS formation, and both NAC and another antioxidant propyl gallate completely rescued cells from cefotaxime-induced cell death (Fig. 2b, c). Moreover, it turned out that salicylate and antimycin as macroautophagy inducers potently suppressed cefotaxime-induced cell death without decreasing ROS levels (Figs. 1f, 2d). These results strongly suggest that ALIS are formed downstream of the cephalosporin-induced ROS production, and that oxidative stress-mediated ALIS formation is responsible for the cephalosporin-induced cell death.

### The stress-activated JNK and p38 MAPK are dispensable for the cephalosporin-induced cell death

**Fig. 3:**
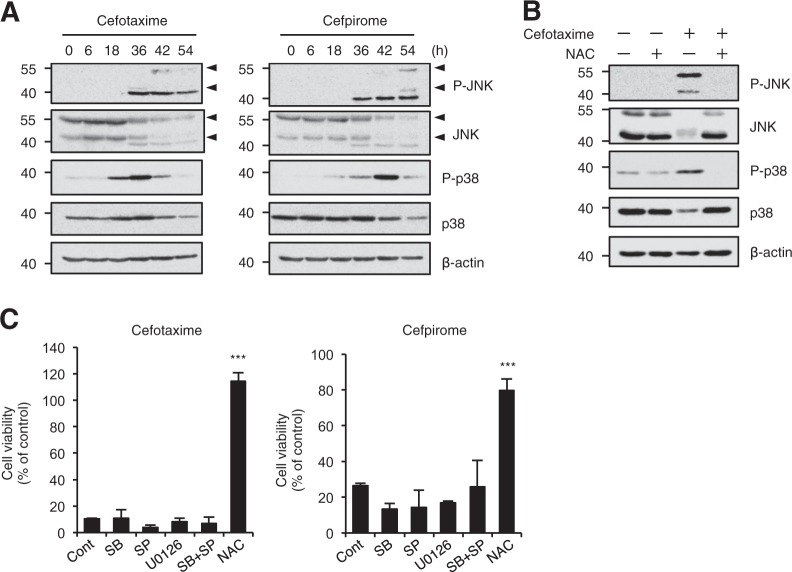
The stress-activated JNK and p38 MAPK are dispensable for the cephalosporin-induced cell death. **a**, **b** HT1080 cells were treated with 1 mg/ml cefotaxime or cefpirome for the indicated periods (**a**) or 36 h (**b**), and then cell extracts were subjected to immunoblotting with the indicated antibodies. Arrow heads indicate appropriate JNK bands. **c** HT1080 cells were treated with 1 mg/ml cefotaxime or cefpirome for 48 h in the presence of various inhibitors; p38 MAPK inhibitor SB203580 (SB, 10 µM), JNK inhibitor SP600125 (SP, 10 µM), ERK inhibitor U0126 (10 µM), and the antioxidant NAC (1 mM), and then subjected to cell viability assay. Data shown are the mean ± SD. Significant differences were determined by one-way ANOVA, followed by Tukey–Kramer test; ***p < 0.001

The stress-activated c-Jun N-terminal kinase (JNK) and p38 mitogen-activated protein kinase (MAPK) play pivotal roles for oxidative stress-mediated cellular responses, including cell death and immune responses^41,42^. We therefore examined whether JNK and p38 MAPK signaling pathways are involved in the cephalosporin-induced cell death. As shown in Fig. 3a, b, JNK and p38 MAPK were robustly activated, even though, for unknown reasons, both total JNK and p38 were reduced in a time-dependent manner, which correlated with the timing of cell death, and co-treatment with NAC completely inhibited the activation. However, the MAPK inhibitors for JNK (SP600125), p38 MAPK (SB203580), and ERK (U0126), failed to suppress the cell death, indicating that the contribution of MAPK signaling pathways to the cephalosporin-induced cell death is relatively small (Fig. 3c).

### p62 is required for the ALIS formation and cell death induced by the cephalosporins

**Fig. 4:**
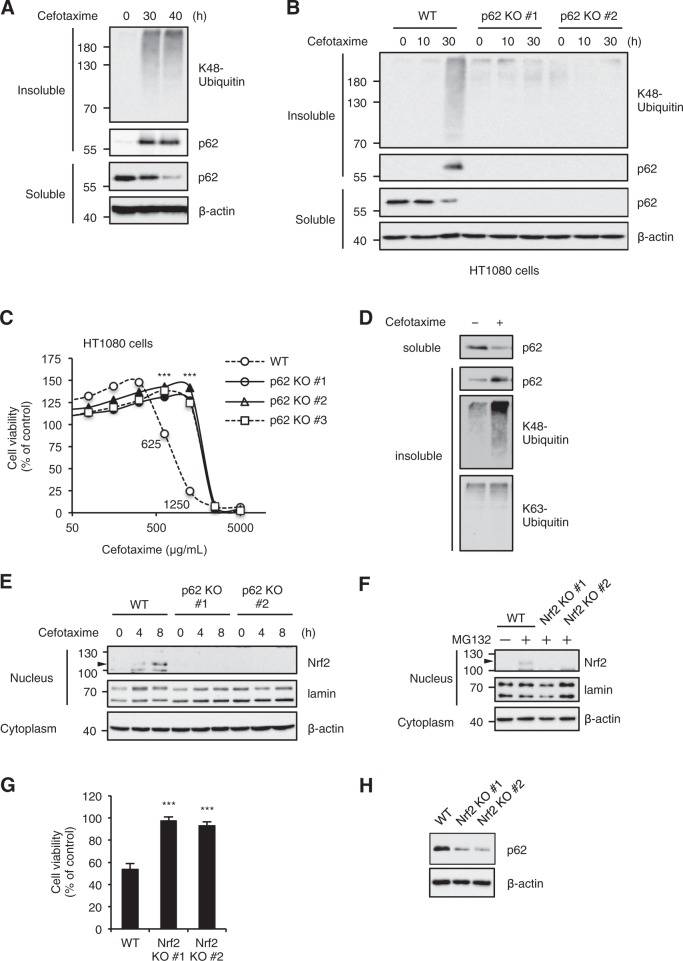
p62 is required for the ALIS formation and cell death induced by the cephalosporins. **a**, **b** HT1080 cells were treated with 1 mg/ml cefotaxime for the indicated periods, and then the detergent-soluble and -insoluble fractions were subjected to immunoblotting with the indicated antibodies. **c** HT1080 cells were treated with the indicated concentrations of cefotaxime or cefpirome for 48 h, and then subjected to cell viability assay. Data shown are the mean ± SD. Significant differences were determined by one-way ANOVA, followed by Tukey–Kramer test; ***p < 0.001. **d** HT1080 cells were treated with 1 mg/ml cefotaxime for 30 h, and then the detergent-soluble and -insoluble fractions were subjected to immunoblotting with K48- and K63-linkage specific polyubiquitin antibodies. **e** HT1080 cells were treated with 1 mg/ml cefotaxime for the indicated periods, and then the nuclear and cytoplasmic extracts were subjected to immunoblotting with the indicated antibodies. **f** After treatment with proteasome inhibitor MG132 for 3 h in order to stabilize Nrf2 protein, the nuclear and cytoplasmic extracts of HT1080 cells were subjected to immunoblotting with the indicated antibodies. **g** HT1080 cells were treated with 1 mg/ml cefotaxime for 48 h, and subjected to cell viability assay. Data shown are the mean ± SD. Significant differences were determined by one-way ANOVA, followed by Tukey–Kramer test; ***p < 0.001. **h** HT1080 cells were subjected to immunoblotting with the indicated antibodies

Recently, several studies have demonstrated that oxidative stress induces the formation of ALIS, which contain K48-linked polyubiquitinated proteins and p62^1,2,6,7^. In agreement with these findings, we observed that cefotaxime promotes the accumulation of p62 in the detergent-insoluble fraction, accompanied by the K48-linked polyubiquitinated proteins (Fig. 4a). Therefore, we examined whether p62 is involved in cefotaxime-induced ALIS formation by using p62 knockout HT1080 cells, which are independently generated by Clustered Regularly Interspaced Short Palindromic Repeat/CRISPR-associated protein-9 nuclease (CRISPR/Cas9) system. As shown in Fig. 4b, the accumulation of the K48-linked polyubiquitinated proteins in the detergent-insoluble fraction was clearly attenuated in p62 knockout HT1080 cells. Moreover, p62 knockout HT1080 cells exhibited significant resistance to cefotaxime-induced cell death at sublethal concentrations between 625 and 1250 μg/mL (Fig. 4c). These observations suggest that p62 is required for the ALIS formation and cell death in HT1080 cells. On the other hand, it has been reported that the ubiquitin-associated (UBA) domain of p62 preferentially binds the K63-linked polyubiquitinated proteins, which is required for the aggresome formation^43^. In addition, previous studies have demonstrated that K48-linked polyubiquitin chains inhibit p62 oligomerization, and disassemble p62 aggregation^44,45^. These previous findings prompted us to investigate whether the K63-linked polyubiquitinated proteins are included in the p62-based ALIS. However, the accumulation of K63-linked polyubiquitinated proteins in the detergent-insoluble fraction in wild-type (WT) cells was not evident when compared to the K48-linked polyubiquitinated proteins (Fig. 4d). We therefore concluded the p62-based ALIS induced by cefotaxime mainly consist of the K48-linked polyubiquitinated proteins.

Nrf2 is widely regarded as an essential regulator of oxidative stress responses^19^. More strikingly, accumulating evidence has demonstrated the plausible functional links between p62 and Nrf2^9^. We therefore investigated the involvement of Nrf2 in p62-mediated cell death induced by cefotaxime. Immunoblot analysis of nuclear extracts revealed that cefotaxime clearly promotes Nrf2 nuclear translocation (Fig. 4e). However, in the absence of p62, the Nrf2 nuclear translocation severely attenuated, suggesting that p62 is required for cefotaxime-induced Nrf2 activation (Fig. 4e). Accordingly, p62 knockout confers resistance to cefotaxime-induced cell death without antioxidant responses mediated by Nrf2. On the other hand, we speculated that Nrf2 protects cells from cefotaxime-induced cell death, and investigated its function using Nrf2 knockout cells validated by immunoblotting (Fig. 4f). Surprisingly, contrary to our expectation, Nrf2 knockout HT1080 cells showed marked resistance to cefotaxime-induced cell death, although in general, Nrf2 protects cells from oxidative stress-induced cell death by exerting its redox activity (Fig. 4g)^46,47^. To explain the paradoxical result, we examined the expression levels of p62 in Nrf2 knockout HT1080 cells, because p62 has been reported as a transcriptional target of Nrf2^48^. Indeed, consistent with previous reports revealing that the p62 expression is severely downregulated in cells from Nrf2 knockout mice, the p62 expression is severely impaired in Nrf2 knockout HT1080 cells (Fig. 4h)^49^. We thus concluded that loss of the p62 expression in Nrf2 knockout HT1080 cells is responsible, at least in part, for the resistance to cefotaxime-induced cell death.

### p62 mediates cefotaxime-induced parthanatos

**Fig. 5:**
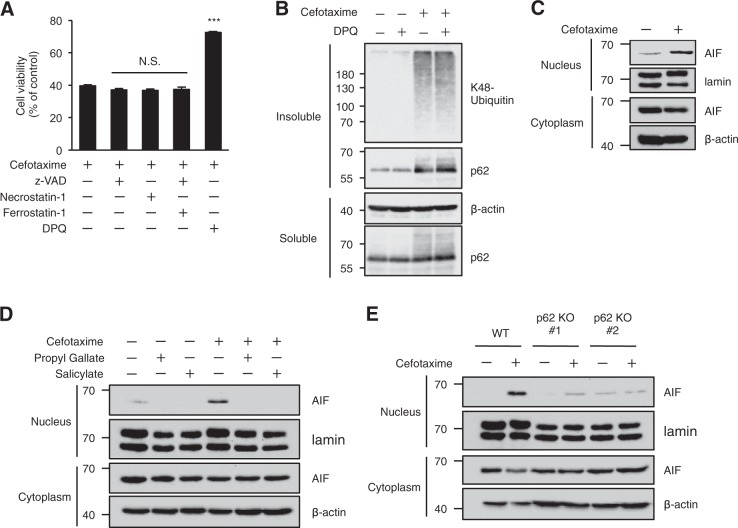
p62 mediates cefotaxime-induced parthanatos. **a** HT1080 cells were treated with the indicated reagents for 48 h, and then subjected to cell viability assay. Data shown are the mean ± SD. Significant differences were determined by one-way ANOVA, followed by Tukey-Kramer test; ***p < 0.001; N.S., not significant. **b** HT1080 cells were treated with the indicated reagents for 24 h. The detergent-soluble and -insoluble fractions were subjected to immunoblotting with the indicated antibodies. **c**–**e** HT1080 cells were treated with the indicated reagents for 48 h, and then the nuclear and cytoplasmic extracts were subjected to immunoblotting with the indicated antibodies. Cefotaxime (1 mg/ml), z-VAD (30 µM), Necrostatin-1 (20 µM), Ferrostatin-1 (10 µM), and DPQ (30 µM), Propyl Gallate (40 µM), Salicylate (10 mM)

Oxidative stress induces different types of cell death, including apoptosis, necroptosis, ferroptosis, and parthanatos depending on cellular context^29,50^. We therefore investigated the types of cell death induced by cefotaxime in HT1080 cells. As shown in Fig. 5a, cefotaxime-induced cell death was significantly suppressed by co-treatment with the PARP-1 inhibitor 3,3′,5,5′-tetra-tert-butyldiphenoquinone (DPQ) that inhibits parthanatos, but not with the pan-caspase inhibitor z-VAD-fmk, Necrostatin-1, and Ferrostatin-1 that inhibits apoptosis, necroptosis, and ferroptosis, respectively. On the other hand, DPQ failed to suppress cefotaxime-induced ALIS formation, suggesting that the ALIS formation occurs upstream of the PARP-1 activation (Fig. 5b). Immunoblot analysis revealed that cefotaxime clearly provokes the translocation of AIF to nucleus, which is widely regarded as a hallmark of the induction of parthanatos (Fig. 5c). As well as inhibiting cefotaxime-induced cell death shown in Figs. 1f, 2c, both propyl gallate and salicylate potently inhibited cefotaxime-induced translocation of AIF to nucleus (Fig. 5d). Moreover, the nuclear translocation of AIF was dramatically attenuated in p62 knockout cells (Fig. 5e). These findings suggest that the p62-based ALIS mediate cefotaxime-induced parthanatos by promoting the nuclear translocation of AIF.

### p62 mediates oxidative stress-induced parthanatos

**Fig. 6:**
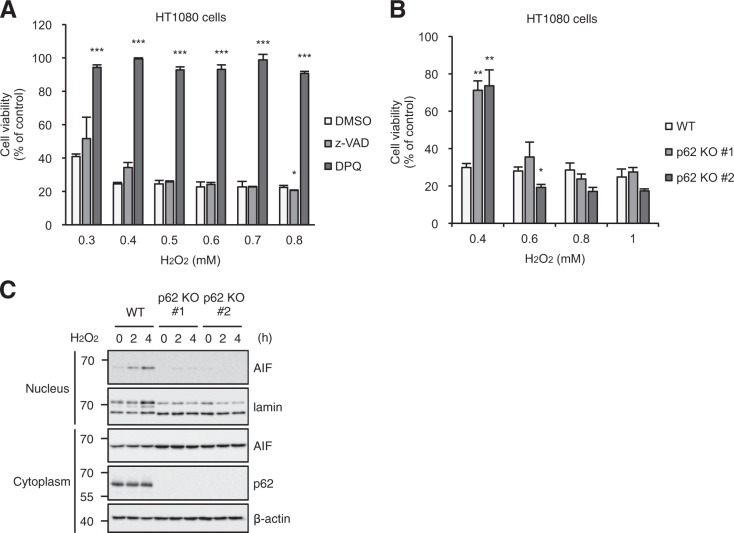
p62 mediates oxidative stress-induced parthanatos. **a**, **b** HT1080 cells were treated with the indicated concentrations of H_2_O_2_ for 5 h, and then subjected to cell viability assay. Data shown are the mean ± SD. Significant differences were determined by one-way ANOVA, followed by Tukey–Kramer test; ***p < 0.001; **p < 0.01; *p < 0.05. **c** HT1080 cells were treated with 0.4 mM H_2_O_2_ for the indicated periods, and then the nuclear and cytoplasmic extracts were subjected to immunoblotting with the indicated antibodies

Given that p62 is required for cefotaxime-induced parthanatos, it is formally possible that p62 is also involved in oxidative stress-induced parthanatos. To explore this possibility, we investigated potential functions of p62 for hydrogen peroxide (H_2_O_2_)-induced parthanatos. When HT1080 cells were exposed to various concentrations of H_2_O_2_, HT1080 cells had significantly decreased cell viability at all concentrations tested, meaning that H_2_O_2_ induces cell death in HT1080 cells (Fig. 6a). Interestingly, co-treatment with DPQ, but not z-VAD-fmk, dramatically inhibited H_2_O_2_-induced cell death, suggesting that H_2_O_2_ preferentially induces parthanatos rather than apoptosis over a broad range of concentrations in HT1080 cells (Fig. 6a). More interestingly, when exposed to lower concentrations of H_2_O_2_, p62 knockout cells were highly resistant to H_2_O_2_-induced parthanatos, whereas there was no difference in higher concentrations (Fig. 6b). These observations suggest that p62 possesses the ability to increase sensitivity to oxidative stress-induced parthanatos. Furthermore, we observed that low concentration (0.4 mM) of H_2_O_2_ clearly induces the nuclear translocation of AIF, which is nearly canceled in p62 knockout HT1080 cells, indicating that p62 is required for the AIF translocation to induce parthanatos (Fig. 6c). From our results, cefotaxime is thought be one of pathophysiological stimulators that generate low concentration of ROS.

### Nuclear accumulation of the p62-based ALIS is required for oxidative stress-induced parthanatos

**Fig. 7:**
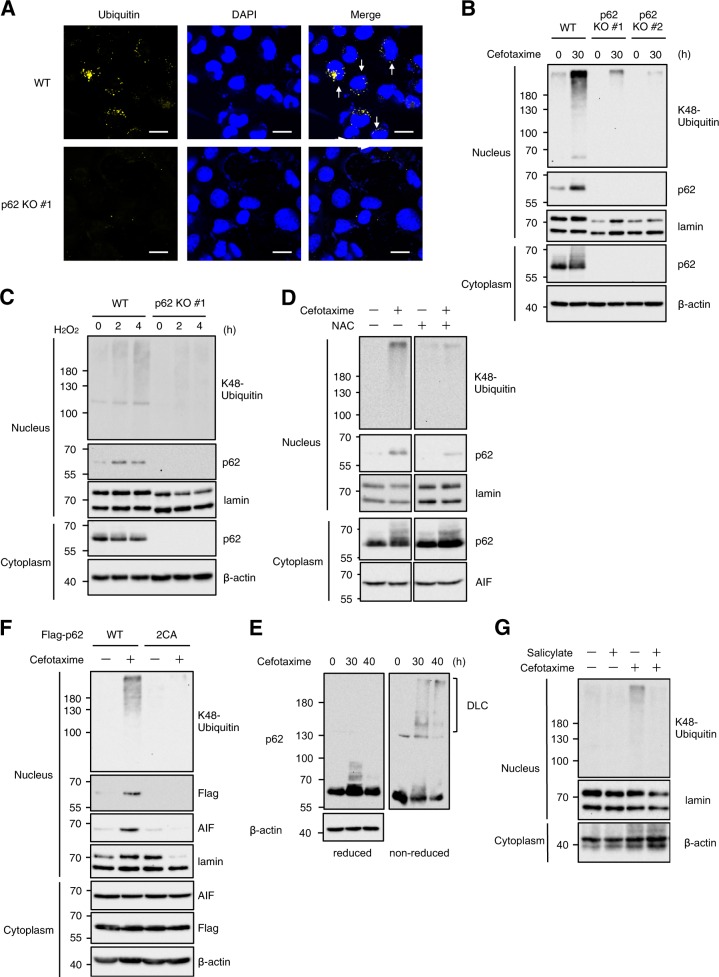
Nuclear accumulation of the p62-based ALIS is required for oxidative stress-induced parthanatos. **a** HT1080 cells were treated with 1 mg/ml cefotaxime for 36 h, and then performed immunofluorescence staining with ubiquitin antibody, and 4′,6-diamidino-2-phenylindole (DAPI) nuclear staining. Arrows indicate the ALIS-containing nucleus. Scale bar, 10 μm. **b**, **c** HT1080 cells were treated with 1 mg/ml cefotaxime (**b**) or 0.4 mM H_2_O_2_ (**c**) for the indicated periods, and then the nuclear and cytoplasmic extracts were subjected to immunoblotting with the indicated antibodies. **d**, **g** HT1080 cells were treated with 1 mg/ml cefotaxime with or without 1 mM NAC (**d**) or 5 mM salicylate (**g**) for 30 h, and then cell extracts were subjected to immunoblotting with the indicated antibodies. **e** HT1080 cells were treated with 1 mg/ml cefotaxime for the indicated periods, and then cell extracts were subjected to immunoblotting under reduced or non-reduced condition. **f** p62 knockout HT1080 cells were transfected with Flag-p62 wild-type (WT) or Flag-p62 C105A/C113A (2CA) plasmid for 24 h, and then treated with 1 mg/ml cefotaxime for 30 h. The nuclear and cytoplasmic extracts were subjected to immunoblotting with the indicated antibodies

In regard to the mechanisms by which p62 mediates oxidative stress-induced parthanatos, we speculated that the p62-based ALIS accumulate in nucleus, because the induction of parthanatos is regulated by a series of nuclear events^29^. In addition, the detergent-insoluble fraction prepared by our procedure includes nuclear proteins. Immunofluorescent staining revealed that cefotaxime-induced puncta formation of ubiquitinated proteins was completely attenuated in p62 knockout HT1080 cells, and as expected, some of the puncta are contained in nucleus when wild-type of HT1080 cells were treated with cefotaxime (Fig. 7a). The nuclear accumulation of the K48-linked polyubiquitinated proteins that depends on p62 was confirmed by immunoblot analysis of nuclear extracts (Fig. 7b, c). Moreover, both cefotaxime and H_2_O_2_ clearly induced nuclear accumulation of p62 (Fig. 7b, c). The nuclear accumulation of p62 was suppressed by co-treatment with NAC, indicating that the p62 accumulation is triggered by oxidative stress (Fig. 7d). Notably, recent evidence has shown a potential function of p62 as a redox sensor^22^. Under oxidative stress conditions, p62 can directly sense ambient redox status by harboring oxidation-sensitive cysteines (Cys105 and Cys113), and forms disulphide bond-mediated p62 oligomers designated disulphide-linked conjugates (DLC), which allow the p62-dependent aggresome formation^22^. Indeed, we observed the high-molecular smear bands, when cell extracts from cefotaxime-treated cells are analyzed by immunoblot analysis under non-reducing conditions, suggesting that DLC formation occurred prior to the ALIS formation (Fig. 7e). Interestingly, we also observed that double substitution of Cys105 and Cys113 apparently blocked the nuclear accumulation of p62 (Fig. 7f). Moreover, the p62 mutant (p62 C105A/C113A) failed to induce nuclear translocation of AIF (Fig. 7f). These observations suggest that the DLC formation-mediated accumulation of p62 in the nucleus is required for oxidative stress-induced parthanatos. In addition, we confirmed that the macroautophagy inducer salicylate clearly inhibited the chemical stress-induced ALIS formation in the nucleus (Fig. 7g). Thus, our findings demonstrate that the formation and nuclear accumulation of the p62-based ALIS play a key role in the induction of parthanatos triggered by oxidative stress.

## Discussion

**Fig. 8:**
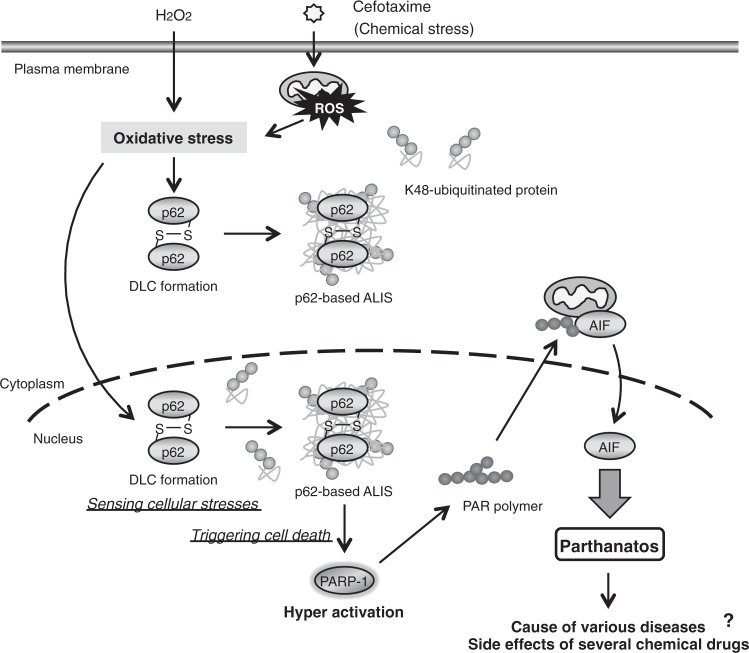
A proposed model for p62-mediated regulation of oxidative stress-induced parthanatos. See the Discussion section

In Fig. 8, a schematic model to explain our study was described. We firstly found that the cytotoxic stress induced by chemical drugs, cephalosporins, including cefotaxime and cefpirome, promotes the formation of cellular microdomains ALIS, and noticed that the ALIS formation correlates well with parthanatos as an atypical type of programmed cell death. Our further studies revealed for the first time that the cephalosporin-induced parthanatos is mediated by oxidative stress, which promotes the ALIS formation, and that the ALIS formation and parthanatos require p62. The p62-based ALIS function as the microdomains sensing cellular stresses, such as oxidative stress. Thus, we identified p62 and the p62-based ALIS as key determinants of oxidative stress-induced parthanatos, which may lead to the cause of various diseases and the side effects of several chemical drugs (Fig. 8).

The cephalosporins are broad-spectrum antibiotics used to control various infectious diseases. Although the principal side effect of the cephalosporins is hypersensitivity especially for penicillin-sensitive patients, the cephalosporins cause rare but serious side effects, such as acute tubular necrosis (ATN) characterized by destruction of tubular epithelial cells^51^. Despite the fact that chemical drugs are one of the major sources of ATN, the pathogenetic mechanisms of the drug-associated ATN remain largely unknown. In this regard, recent reports have demonstrated that the bactericidal antibiotics cause oxidative stress-mediated cell death through mitochondrial dysfunction^39,40^. We therefore hypothesized, and as expected, found that the cephalosporins cause ROS generation that leads to cell death. Interestingly, we also found that the cephalosporins preferentially induce parthanatos mediated by the p62-based ALIS. Considering that macroautophagy inducers, such as salicylate and antimycin, strongly suppressed both the ALIS formation and parthanatos without decreasing ROS levels as shown in Figs. 1e, 2d, cephalosporin-induced ROS generation is not lethal, and the p62-dependent ALIS formation downstream of ROS generation is essential for the induction of parthanatos. Accordingly, the ALIS function as a switch to induce parthanatos, and accumulation of the ALIS may determine the sensitivity to stress-induced cell death. Thus, although further studies are needed to elucidate the involvement of the p62-based ALIS in the cephalosporin-induced destruction of tubular epithelial cells, our results may provide insight into the pathogenetic mechanisms of the cephalosporin-induced ATN, as one of stress-triggered tissue damage and diseases, by which cephalosporins enriched in the urine undergo massive oxidative stress, leading to tubular epithelial cell parthanatos mediated by the p62-based ALIS.

In dendritic cells (DCs), microbial products, such as LPS, promote the ALIS formation, so-called DALIS (dendritic cell aggresome-like induced structures), to sequester polyubiquitinated defective ribosomal products (DRiPs) prior to their clearance by the proteasome^52,53^. Interestingly, DCs seem to intentionally increase DRiPs in order to promote the DALIS formation, and then enhance the ability to control MHC class I loading and peptide presentation by employing DALIS as antigen storage compartments^52^. Although ALIS are conceived as storage for polyubiquitinated proteins prior to their degradation, this example in DCs has shown a possibility that ALIS are required to elicit appropriate physiological responses. In the present study, we demonstrate that the p62-based ALIS induced by oxidative stress act as the microdomains that play an important role in oxidative stress-induced parthanatos. The unexpected and novel function of ALIS may provide an explanation for the toxic effects of protein aggregates observed in a wide variety of diseases, including human neurodegenerative diseases. Moreover, our findings implicate the p62-based ALIS in oxidative stress-induced parthanatos. Since ALIS, p62, oxidative stress, and parthanatos have been individually associated with human neurodegenerative diseases, our results may demonstrate biologic relationships among them, which might be crucial for understanding the pathophysiological basis of human neurodegenerative diseases.

p62 is a multifunctional protein that mediates a broad range of cellular responses, including autophagy, antioxidant responses, and apoptosis^9^. In this study, we show that oxidative stress promotes p62-dependent ALIS formation and its accumulation to nucleus, which mediates oxidative stress-induced parthanatos. Based on our results shown in Fig. 7e, f, the oxidation and oligomerized DLC formation of p62 are thought to be critical for the ALIS formation and nuclear accumulation. In particular, oxidation-sensitive cysteines (Cys105 and Cys113) of p62 seem to play an important role in these molecular dynamics by sensing oxidative stress and forming disulphide bond-mediated oligomers. However, precise mechanisms by which oxidative stress promotes the p62 accumulation in nucleus remain challenging. It has shown in a previous report that two nuclear localization signals (NLS) located in the N-terminal and C-terminal of p62 allow the nuclear-cytoplasmic shuttling of p62, which contributes to the assembly of proteasome-containing degradative compartments in nucleus^54^. Taking into consideration that the nuclear-cytoplasmic shuttling of p62 is modulated by phosphorylations near the C-terminal NLS of p62, the phosphorylation-dependent mechanisms may be linked to oxidative stress-dependent nuclear translocation of the ALIS^54^. Identifying the responsible kinases is a key process for understanding the regulatory mechanisms of nuclear translocation of the p62-based ALIS. Meanwhile, it seems likely that high molecular weight protein aggregates, such as the p62-based ALIS, can not translocate to the nucleus. Nuclear autophagy that has emerged as a novel mechanism of degradation of nuclear aggregates may be another possibility to explain the p62 accumulation in nucleus^55^. In steady-state condition, nuclear autophagy keeps the p62 expression low, but oxidation-dependent conformational change of p62 makes it possible to escape from nuclear autophagy, resulting in accumulation of the p62-based ALIS formation. In any case, future research will uncover the molecular basis for the nuclear accumulation of p62.

Finally, the most important issue is how the p62-based ALIS activate PARP-1 and subsequently regulate nuclear translocation of AIF that determines the induction of parthanatos. PARP-1 is activated by severe genomic stress and generates poly (ADP-ribose) polymer, which stimulates the nuclear translocation of AIF (Fig. 8). p62 has been reported to interact with DNA repair factors, such as the ubiquitin E3 ligase RNF168^56^. The p62-based ALIS may act as the microdomains trapping and inhibiting the DNA repair factors, leading to exacerbation of genomic stress, which causes hyperactivation of PARP-1. To answer the question, we need to explore the nuclear events evoked by the p62-based ALIS under oxidative stress conditions.

Thus, although further studies are required for the elucidation of the mechanisms by which the p62-based ALIS induce parthanatos, our results uncovered novel functions of p62 and the p62-based ALIS that stimulate oxidative stress-induced parthanatos, which highlights the importance of p62 and the p62-based ALIS as stress sensors and critical determinants of life and death decisions in oxidative stress response. Our future studies will provide completely new insight into the pathogenesis of the stress-induced tissue damage and diseases.

## Materials and methods

### Reagents and plasmids

Cefotaxime Sodium Salt, Cefpirome Sulfate, Cephalexin, SP600125, NAC, salicylate and H_2_O_2_ were purchased from Wako. Propyl gallate, Ferrostatin-1 and U0126 were purchased from Sigma. z-VAD-fmk was purchased from Peptide Institute. SB203580, antimycin, apocynin, Necrostatin-1, DPQ and Mito-TEMPO were purchased from Santa Cruz. The antibodies used were against phospho-p38, p38, phospho-JNK, JNK, K48- and K63-ubiquitin, AIF and Lamin A/C (Cell Signaling), p62 (MBL), and β-actin (Santa Cruz). cDNAs encoding human p62 was obtained by performing PCR, and was inserted into pcDNA3 with Flag tag plasmid. Flag-p62 C105A/C113A mutant was generated by mutating ^105^Cys and ^113^Cys to Ala in p62. Plasmid transfection was performed using Polyethylenimine “Max” (PEI-MAX, Polysciences), according to the manufacturer’s instructions.

### Cell lines

HT1080 cells were grown in Dulbecco’s Modified Eagle Medium (DMEM), 10% heat-inactivated fetal bovine serum (FBS), and 1% penicillin-streptomycin solution, at 37 °C under a 5% CO_2_ atmosphere.

### Colorimetric cell viability assay

Cells were seeded on 96-well plates. After indicated stimulation or treatment, cell viability was determined using Cell Titer 96 Cell Proliferation Assay (Promega), according to the manufacturer’s protocol. The absorbance was read at 492 nm using a microplate reader. Data are normalized to control (100%) without stimulus, unless noted otherwise.

### Immunoblot

Cell extracts were separated into detergent-soluble and -insoluble fractions with the 1% Triton X-100 buffer [20 mM Tris-HCl (pH 7.4), 150 mM NaCl, 1% Triton-X100, 10% Glycerol, and 1% protease inhibitor cocktails (Nacalai Tesque)]. The detergent-insoluble fraction was solubilized in the 1% Triton X-100 buffer supplemented with 1% SDS and benzonase (Sigma). Both the detergent-soluble and -insoluble fractions were subjected to immunoblot analysis as previously described^57^.

### Bioimaging and quantification of ROS

HT1080 cells were seeded on glass plates. After stimulation, cells were treated with 10 µM DCFH-DA for 30 min at 37 °C. After washing with PBS, the intracellular ROS generation was observed using a Zeiss LSM800 laser confocal microscope (Carl Zeiss) and the images were processed with Zen software. The fluorescence images were obtained from three deferent fields of view. Data shown are the mean ± SD of three images.

### Generation of knockout cell lines

*p62* (*SQSTM1*) and *Nrf2-* knockout cells were generated using the CRISPR/Cas9 system^58,59^. Guide RNAs (gRNAs) were designed to target a region in the exon 3 of *p62* gene (5′-AGACTACGACTTGTGTAGCG-3′), and *Nrf2* gene (5′- CTGGGCTCTCGATGTGACC-3′) using CRISPRdirect^60^. gRNA-encoding oligonucleotide was cloned into lentiCRISPRv2 plasmid (*p62*) or gRNA cloning vector (*Nrf2*) (addgene)^61^, and knockout cells were established as previously described^62^. To determine the mutations of *p62* in cloned cells, genomic sequence around the target region was analyzed by PCR-direct sequencing using extracted DNA from each clone as a template and the following primers: 5′-CACCGCGCTACACAAGTCGTAGTCT-3′and 5′-AAACAGACTACGACTTGTGTAGCGC-3′.

### Immunofluorescence staining

HT1080 cells were fixed with 3.7% formaldehyde, permeabilized with 0.5% Triton X-100, blocked with 3% BSA-PBS, and incubated with primary antibodies (anti-ubiquitin) overnight at 4 °C, followed by incubation with secondary antibodies (goat anti-mouse Alexa Fluor 555, Invitrogen) for 1 h at room temperature. The immunostained samples were enclosed with Fluoro-KEEPER Antifade Reagent, Non-Hardening Type with DAPI (Nakalai), and observed using a Zeiss LSM800 laser confocal microscope.

### Nuclear extraction

Cells were lysed in ice-cold lysis buffer containing 10 mM HEPES (pH 7.5), 10 mM KCl, 0.1 mM EGTA, 0.1 mM EDTA, 1 mM DTT, and 1% protease inhibitor cocktails (Nacalai Tesque) for 15 min. Cell lysates were added 1% NP-40, and then centrifuged at 4 °C at 2,500 rpm for 3 min. After the supernatants containing cytoplasmic fraction were removed, the pellets were suspended in ice-cold lysis buffer containing 20 mM HEPES (pH 7.5), 400 mM NaCl, 1 mM EGTA, 1 mM DTT, and 1% protease inhibitor cocktails for 15 min with vortexed every 5 min. Cell lysates were then centrifuged at 4 °C at 15,000 rpm for 15 min, and then the supernatants were collected as nuclear fractions.
